# Effectiveness of a Lifestyle Intervention on Social Support, Self-Efficacy, and Physical Activity among Older Adults: Evaluation of *Texercise* *Select*

**DOI:** 10.3390/ijerph15020234

**Published:** 2018-01-30

**Authors:** Marcia G. Ory, Shinduk Lee, Gang Han, Samuel D. Towne, Cindy Quinn, Taylor Neher, Alan Stevens, Matthew Lee Smith

**Affiliations:** 1 Center for Population Health and Aging, Texas A&M University, College Station, TX 77843, USA; sduklee@sph.tamhsc.edu (S.L.); towne@sph.tamhsc.edu (S.D.T.J.); quinn@sph.tamhsc.edu (C.Q.); matthew.smith@tamhsc.edu (M.L.S.); 2 Department of Environmental and Occupational Health, School of Public Health, Texas A&M University, College Station, TX 77843, USA; 3 Department of Health Promotion and Community Health Sciences, School of Public Health, Texas A&M University, College Station, TX 77843, USA; 4 Department of Epidemiology and Biostatistics, School of Public Health, Texas A&M University, College Station, TX 77843, USA; ghan@sph.tamhsc.edu; 5 Southwest Rural Health Research Center, Texas A&M University, College Station, TX 77843-1266, USA; 6 College of Public Health, University of Arkansas, Little Rock, AR 72205, USA; tlneher@uams.edu; 7 Center for Applied Health Research, Baylor Scott & White Health, Temple, TX 76502, USA; Alan.Stevens@BSWHealth.org; 8 Department of Health Promotion and Behavior, College of Public Health, The University of Georgia, Athens, GA 30602, UGA

**Keywords:** lifestyle intervention, evidence-based programs, healthy aging, physical activity, program evaluation

## Abstract

Despite the well-recognized benefits of physical activity across the life course, older adults are more inactive than other age groups. The current study examines the effects of *Texercise Select* participation on self-reported sedentary, light, moderate, and vigorous physical activity. Secondarily, this study examined intervention effects on two potential facilitators of physical activity: (1) self-efficacy for being more physically active and (2) social support received for physical activity. This study used a non-equivalent group design with self-reported surveys administered at baseline, three-month (immediate post for cases) and six-month follow-ups for the intervention (*n* = 163) and a comparison group (*n* = 267). Multivariable mixed model analyses were conducted controlling for age, sex, race, ethnicity, education, comorbid conditions, and site. Among the intervention group, the program had significant immediate effects on most primary outcomes (*p* < 0.05) at three months. Furthermore, significant improvements were observed for all physical activity intensity levels at six months (*p* < 0.05). The reduction in sedentary behavior and increases in all physical activity intensity levels were significantly greater from baseline to three-month and baseline to six-month follow-ups among intervention group participants relative to those in the comparison group. This study confirms the effectiveness of *Texercise Select* to reduce sedentary behavior and improve physicality, supporting the intervention’s robustness as a scalable and sustainable evidence-based program. It also counters negative stereotypes that older adults are not interested in attending multi-modal lifestyle intervention programs nor able to make health behavior changes that can improve health and overall functioning.

## 1. Introduction

There is a growing body of evidence documenting the positive health impacts of physical activity at all ages [1,2]. The classic US Department of Health and Human Services Physical Activity Guidelines for Americans [1] recognizes the continued health benefits of regular physical activity among the rapidly growing older population [3]. In fact, national physical activity guidelines are typically the same for adults and older adults who have no limiting chronic conditions. Some debate remains about the exact amount and type of physical activity that are most appropriate for different individuals with specific conditions [4]. However, as indicated in the guidelines, avoiding inactivity is universally recommended across the life course. The recommendation of at least 150 min of weekly moderate-intensity physical activity (e.g., brisk walking) persists for most older adults. Further, recommendations to engage in vigorous-intensity activity (e.g., jogging, swimming laps) are not restricted based on age. The guidelines recognize special circumstances relevant to some older adults and include qualifications that older adults should be as physically active as their abilities and conditions allow.

The health-enhancing effects of physical activity on most physical, mental, and cognitive conditions are especially-relevant for older Americans who typically have one or more chronic conditions [2,5,6,7]. Yet, older adults are among the least physically active population group in America [8] despite their “general knowledge” about the importance of physical activity [9]. Studies have examined different determinants of physical activity in later life [10,11]. Physical activity among older adults is influenced by multiple factors including self-efficacy or skills to integrate physical activity into daily life, social support, financial resources for physical activity, and knowledge about physical activity programs [12,13]. According to a systematic literature review of physical activity and exercise interventions, change in self-efficacy was positively associated with participation in exercise among older adults [10]. Another systematic review reported a positive association between social support for physical activity and engagement in leisure time physical activity among older adults [14]. Given the complexity associated with behavior in a real-world setting, interventions with cognitive and behavioral components can improve participants’ initial and sustained outcomes [12]. Fortunately, over the past two decades, there has been a proliferation of multi-modal evidence-based programs to promote physical activity among older adults to better manage chronic conditions and/or reduce fall risks [15,16,17,18,19]. Many of these programs were generated from highly-controlled research studies, and then translated for more wide-spread delivery into community or clinical settings. As a result, program scalability and sustainability has often proved challenging [20,21,22,23].

An exception is *Texercise Select*, which grew out of a Governor’s Office health promotion initiative [24]. The original version, *Texercise Classic*, is a 12-week health promotion program targeting physical activity and healthy diet [24]. *Texercise Classic* experienced early successes in program scaling and sustainability because of strong stakeholder support at the local and state-wide level [25]. Unlike many other evidence-based programs [15,16,17,18,19], *Texercise Classic* is loosely-structured and offers substantial flexibility to its participants and leaders in terms of the program delivery (e.g., type and amount of exercise/physical activity) [24]. Despite its early success, *Texercise Classic* is not viewed as an evidence-based program because the program was not delivered uniformly across groups. The lack of standardization also made process and summative program evaluations difficult [24]. Thus, *Texercise Select* was developed as a standardized lifestyle program (i.e., facilitators deliver the intervention using a manual containing session-by-session content and activity guides) to ensure more consistent implementation and evaluation [24]. For example, while *Texercise Classic* does not specifically recommend particular exercises for participants, *Texercise Select* has a prescribed list of exercises for flexibility, strength, balance, and endurance that account for about 30–45 min of each session [24]. Drawing from social cognitive learning concepts, *Texercise Select* was designed to enhance self-efficacy and behavioral skills and thereby promote and sustain participants’ healthy aging activities [24].

The original *Texercise Select* research study demonstrated positive impacts on increased physical activity [26]. While promising, its findings were based on a pre-post, single-group design. Findings from such designs are considered limited due to threats to internal validity caused by uncontrolled confounding factors potentially influencing study outcomes.

Utilizing a more rigorous study design, the primary purpose of the current study was to explore the effects of *Texercise Select* participation on self-reported sedentary, light, moderate, and vigorous physical activity over time. Secondarily, this study examined intervention effects on two potential indicators of physical activity: (1) self-efficacy for being more active; and (2) social support received for physical activity. We hypothesize that more intervention benefits will be observed among *Texercise Select* participants relative to their comparison group counterparts, and that these gains will be sustained for at least six-months.

## 2. Materials and Methods 

The current study employed a non-equivalent group design with pre- and post-tests for the intervention (i.e., exposed to *Texercise Select*) and comparison (i.e., not exposed to *Texercise Select*) group. For recruitment, the research team partnered with multiple Texas organizations in the aging and health care sectors with organizations that deliver health and wellness programs to older adults. These partners helped identify the study sites, and the participating sites were designated as either intervention or the comparison group. 

There was no random assignment in this pragmatic study approach. The participating sites communicated with the research team about their needs and preference to be in the intervention or comparison group. The delivery of *Texercise Select* served as the distinguishing marker for classification as either the ‘intervention’ or ‘comparison’ group. To address potential selection bias based on this purposeful study design, the research team attempted to recruit similar participants in the intervention and comparison group, and to further minimize any significant differences through adjusted analyses. While the comparison group was not exposed to *Texercise Select*, they were not restricted from participating in other health or exercise programs, and hence could be considered comparable to a “usual care” group. The participating sites involved multiple community settings including senior centers (*n* = 4), senior housing (*n* = 2), an all-purpose community center (*n* = 1), a lifetime education facility (*n* = 1), and a faith-based facility (*n* = 1). As an incentive for participation, the research team agreed to make *Texercise Select* or a comparable evidence-based program available to comparison sites after the six-month study period. 

*Texercise Select* workshop delivery and study-related data collection occurred between May 2015 and September 2017. Once a site was identified as either an intervention or comparison site, the study participants were recruited by the participating sites. The data was collected by the research team so that assessment would be independent from program delivery. The research team supported the recruitment by providing financial incentives and recruitment materials (e.g., hand-outs) to the participating sites. Study participants were invited to complete surveys at baseline, three months, and six months. Study participants received a $25 gift card upon completion of all three rounds of surveys. The study was approved by the Texas A&M University Institutional Review Board IRB2015-0024D. 

### 2.1. Intervention

*Texercise Select* is a lay-led group-based lifestyle enhancement program delivered in a face-to-face workshop format [24]. Typically facilitated by two trained lay leaders, *Texercise Select* includes 20 educational sessions (approx. 1.5 h per session) delivered over the course of approximately 12 weeks. The recommended number of participants per workshop is between 15 and 20. The program incorporates educational components and interactive discussions to promote initiation, engagement, and maintenance of physical activity. Further, after the introductory week, participants engage in structured physical activity exercises (i.e., flexibility, strength, balance, and endurance) drawn from a prescribed list with the goal of 30–45 min of exercise per session. 

Based on the social cognitive learning principles, it is hypothesized that the multiple features of *Texercise Select* help its participants to initiate, engage, and maintain healthy behaviors. Thus, the entire program was designed around the concept of self-efficacy and encouraging participants to take a more active role in their health. Being organized around a “Ready, Set, Go, Stay” rubric, *Texercise Select* promotes healthy aging behaviors (e.g., physical activity) by guiding its participants to practice correct and safe exercises and use skills, such as goal setting, action plans, and problem-solving, and providing social support. 

Following the detailed implementation manuals and material, lay-leader training was provided in a face-to-face group-setting. The following were stressed during the six-hour training: (1) value of and criteria for evidence-based programs along with expectation of adhering to fidelity; (2) explanation of session format with icons and agendas; (3) explanation and demonstration of core self-management practices such as action planning and problem solving; and (4) demonstration of strength, balance, and flexibility exercises. More detailed information about the *Texercise Select* program and lay-leader training is available elsewhere [24].

### 2.2. Study Participants and Procedures

**Figure 1:**
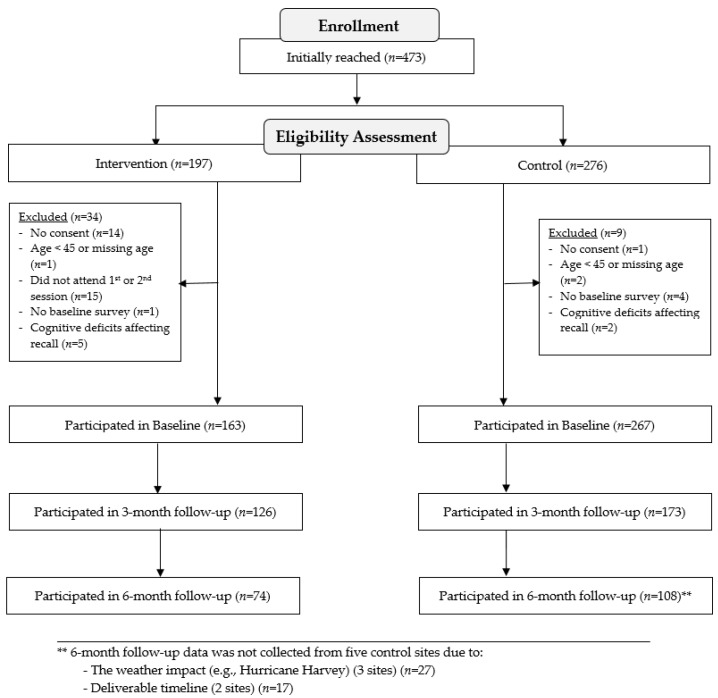
Consort diagram.

For intervention and comparison groups, participants were included in the study if they met the following inclusion criteria: (1) were at least 45 years old; (2) consented to participate in the study; and (3) completed the baseline survey. Although the program developers permit adults 45 and older to join Texercise, for the purposes of this study, adults 60 and older were the focal target age group. Additional inclusion criteria for the intervention group was to have attended the first or second *Texercise Select* workshop session. Based on ethical considerations, and to accommodate the community partners’ needs, the study-ineligible individuals were still allowed to participate in the workshops, although their data were excluded from analyses. Surveys were collected from participants at baseline, three months (immediate post intervention for cases), and six months included self-reported physical activity measures, self-efficacy related to engaging in physical activity, and perceived social support for physical activity. Figure 1 shows the phases of recruitment and data collection.

Based on a public health approach, the program was designed to be safe for community-dwelling older adults across a range of health statuses. Thus, the study did not limit participation based on the participants’ physical health and was open to all interested in becoming more physically active. During program recruitment, the *Texercise Select* program was described to the participants, and a general informational sheet about the study was distributed. All *Texercise Select* lay leaders (facilitators) were required to receive a formal training session about how to safely introduce exercises to the participants. The training sessions were supplemented with course material that included screening questions (e.g., Exercise and Screening for You, EASY [27,28,29]) and safety tips for participants. Furthermore, during the first active session of the program, the study participants are educated about the importance of warm-up and cool-down. The identification of participants’ comfortable exercise levels during the first active session facilitates practicing exercises safely and correctly throughout the program.

### 2.3. Primary Outcome Measures

#### 2.3.1. Physical Activity

Three measures of physical activity and one measure of physical inactivity representing different intensity levels were collected. Physical inactivity or sedentary behavior was measured by the number of hours engaged in sedentary behaviors (e.g., sitting) on a typical weekday modified from the International Physical Activity Questionnaire (IPAQ) [30]. A sequential item tailored from the IPAQ [30] assessed the total number days and minutes per day spent engaging in light (e.g., casual or leisurely walking), moderate (e.g., brisk walking), and vigorous (e.g., running) physical activities. The days and minutes were combined to calculate a total number of minutes of physical activity per week for each physical activity level. For the intervention group, workshop attendance by session was recorded by the lay-leaders.

#### 2.3.2. Self-Efficacy

Six four-point Likert items, developed by the research team for this intervention, were used to measure perceived self-efficacy related to engaging in physical activity. Participants were asked to rate their degree of confidence based on the following: (1) setting a physical activity goal; (2) exercising safely at home; (3) being physically active safely in the neighborhood; (4) identifying outdoor places near their home for engaging in physical activity; (5) identifying physical activities that do not require special equipment or facilities; and (6) identifying facilities near their home for engaging in physical activity. The response categories for these items were “strongly agree,” “agree,” “disagree,” and “strongly disagree”. The composite score could range from 6 to 24, with higher scores indicating greater self-efficacy. The internal reliability for this scale was high for the comparison (Cronbach’s alpha = 0.90) and intervention (Cronbach’s alpha = 0.87) groups.

#### 2.3.3. Social Support

Four four-point Likert scale items, developed by the research team for this intervention, were used to measure the perceived social support for physical activity. Participants were asked to report how frequently they reported getting social support for: (1) planning physical activity goals; (2) keeping physical activity goals; (3) reducing barriers to physical activity; and (4) engaging in physical activity). The response categories for these items were “never,” “rarely,” “sometimes,” and “often”. The composite score could range from 4 to 16, with higher scores indicating greater social support available. The internal reliability for this scale was high for both comparison and intervention groups (Cronbach’s alpha = 0.79).

#### 2.3.4. Other Covariate Measures

The baseline survey included sociodemographic information (e.g., age, sex, race, ethnicity, and education), whether a participant lived alone, and types of chronic conditions (e.g., arthritis/rheumatic disease, breathing/lung disease, cancer, depression or anxiety disorder, diabetes, heart disease, hypertension, stroke, and other). Participants were determined to have comorbid conditions if they reported having two or more of the listed chronic condition types. Due to lack of variability among study participants, race/ethnicity was collapsed into two categories (i.e., non-Hispanic White or other). Three levels of education were included in analyses (i.e., high school graduate or lower education, some college or technical school, or college graduate or higher). 

### 2.4. Statistical Analyses

**Figure 2:**
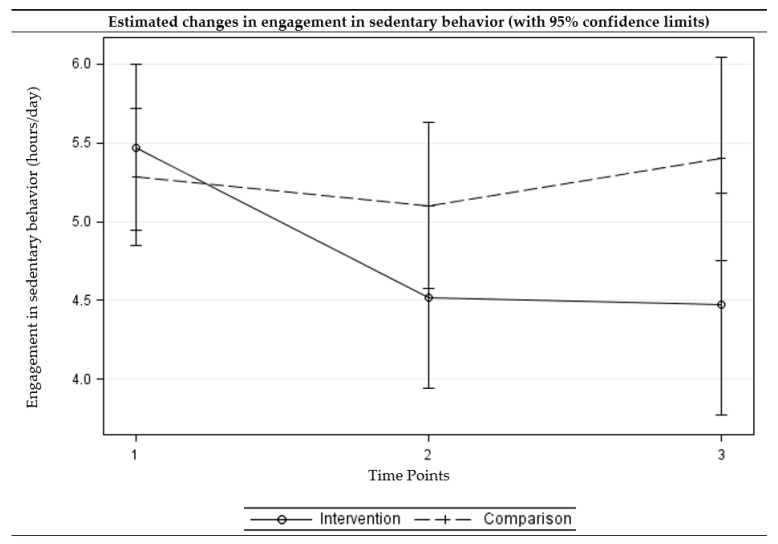
Estimated changes over time in engagement in sedentary behaviors among intervention and comparison groups. (Time points: 1 = baseline, 2 = three-month follow-up, and 3 =six-month follow-up) (Multi-level multivariable mixed-effects linear model).

**Figure 3:**
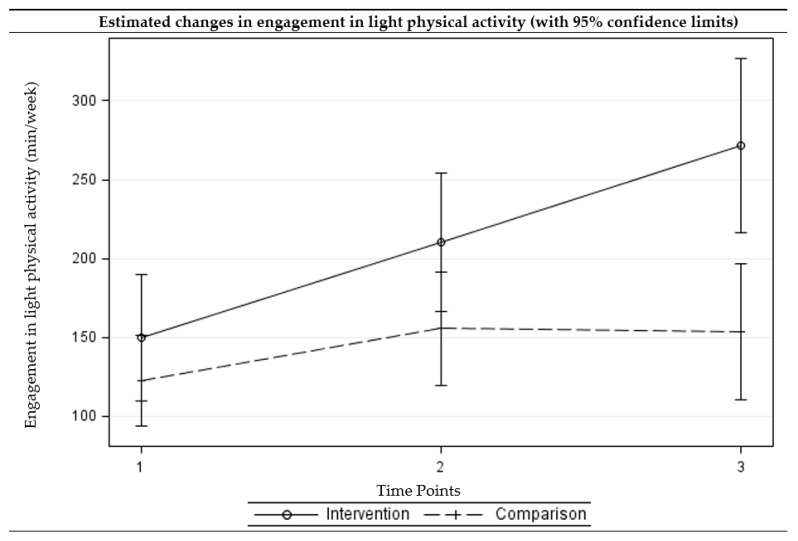
Estimated changes over time in engagement in light physical activities among intervention and comparison groups. (Time points: 1 = baseline, 2 = three-month follow-up, and 3 = six-month follow-up) (Multi-level multivariable mixed-effects linear model).

**Figure 4:**
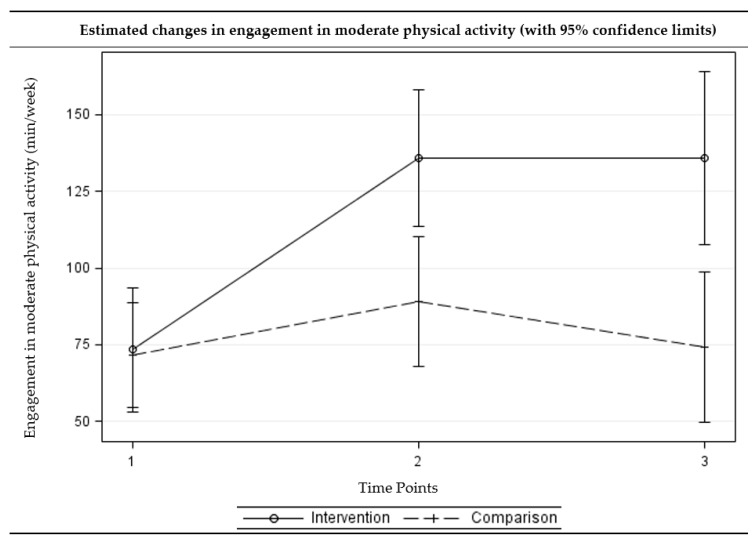
Estimated changes over time in engagement in moderate physical activities among intervention and comparison groups. (Time points: 1 = baseline, 2 = three-month follow-up, and 3 = six-month follow-up) (Multi-level multivariable mixed-effects linear model).

**Figure 5:**
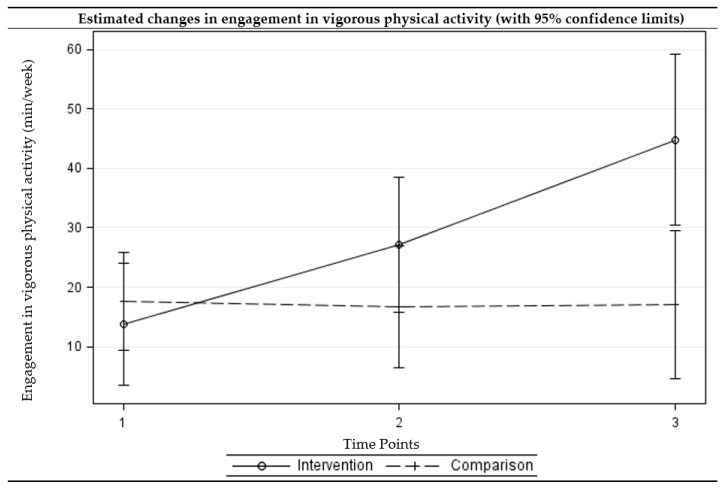
Estimated changes over time in engagement in vigorous physical activities among intervention and comparison groups. (Time points: 1 = baseline, 2 = three-month follow-up, and 3 = six-month follow-up) (Multi-level multivariable mixed-effects linear model).

The study participants were described using means (standard deviation (SD)) for interval variables, frequency (%) for categorical variables, and medians (interquartile range) for highly-skewed interval variables (Table 1). Bivariate analyses (Chi-square test for categorical variables, independent-sample *t*-tests for interval variables, and Wilcoxon rank-sum test for highly-skewed interval variables) were conducted to compare baseline participant characteristics between the intervention and comparison groups (Table 1). Next, the attrition analyses were performed, separately for the intervention and comparison groups, by performing bivariate analyses for comparing participant characteristics (e.g., sociodemographic characteristics, chronic comorbidity, and baseline measures of health behavior) between the study-eligible participants and those who were lost-to-follow-up (Table not shown). Pairwise *t*-tests were used to compare unadjusted changes in all the primary outcome variables, except for vigorous physical activity, from the baseline to three months and baseline to six months, separately for comparison and intervention groups (Table 2). Given its high skewness, a Wilcoxon signed-rank test was used to examine unadjusted changes in vigorous physical activity. In addition, the percent improvement in the primary outcome variables from the baseline to three months and baseline to six months was calculated for all study outcomes. As appropriate, Cohen’s d coefficients were calculated to provide effect sizes for changes from baseline to three months and baseline to six months. Lastly, a series of multi-level multivariable mixed-effects linear models were used to identify changes in primary outcomes from baseline to three months and baseline to six months (Table 3 and Figure 2, Figure 3, Figure 4 and Figure 5). The random-effects from sites were incorporated in the models, and random-intercepts were used. Given the non-linear trend of the outcomes over time, time was classified as a categorical variable instead of a continuous variable. All models included the group (i.e., intervention and comparison groups), time (baseline, three months, and six months), and the interaction between the group and time, and were controlled for age, sex, race, ethnicity, education, and comorbid conditions.

## 3. Results

### 3.1. Study Participants

A total of 430 study-eligible participants were recruited (Figure 1). Of the 430 study-eligible participants, 163 were in the intervention group and 267 were in the comparison group. Of the 163 study-eligible intervention group participants, 126 (77.3%) completed the three-month assessment and 74 (45.4%) completed the six-month assessment. Of the 267 study-eligible comparison participants, 173 (64.8%) completed the three-months assessment and 108 (40.4%) completed the six-months assessment. Among the intervention group, those who remained in the study engaged in less sedentary behaviors (5.4 vs. 6.9 h per day, *p* = 0.019) and more light-level physical activities (172.6 vs. 102.6 min per week, *p* = 0.011) at baseline compared to those who did not complete the three-month assessment. Among the comparison group, no statistically-significant difference was observed between participants with and without three-month or six-month assessments.

The average age of the study-eligible participants was 74 years (ranged from 45 to 98 years; 95% CI = 73.12, 75.44) for the comparison group and 75 years (ranged from 60 to 91 years; 95% CI = 73.65, 76.03) for the intervention group (Table 1). On average, study-eligible intervention participants attended approximately 15 out of 20 sessions, and study-eligible intervention participants with matched baseline and three-month follow-up assessment attended about 17 out of 20 sessions. The intervention and comparison groups were significantly different at baseline in terms of race and ethnicity with the intervention group containing more non-Hispanic White participants (67.7% vs. 34.7%, *p* < 0.001) (Table 1). Participants in the intervention group also reported significantly greater self-efficacy for physical activity (18.5 vs. 17.7 out of 24, *p* = 0.022) and more light-level physical activity (156.0 vs. 118.8 min-per-week, *p* = 0.047) (Table 1).

### 3.2. Program Effects (Unadjusted)

Table 2 presents the descriptive statistics for those with paired data and the results from the unadjusted pairwise comparisons. Statistically significant improvements in social support (*d* = 0.48, *p* < 0.001) and self-efficacy (*d* = 0.33, *p* = 0.008) related to physical activity were observed among the intervention group at three months. While the comparison group also showed statistically significant improvement in social support for engaging in physical activity (*d* = 0.16, *p* = 0.032) at three months, the magnitude of change (*d*) observed among the comparison group was smaller than the change observed among the intervention group. A larger proportion of participants in the intervention group showed improvements in social support (58.8% vs. 45.1%) and self-efficacy (49.2% vs. 37.7%) related to physical activity compared to the comparison group. 

The intervention group reported greater engagement in moderate physical activity (128.5 vs. 70.7 min per week, *d* = 0.53, *p* < 0.001) and vigorous (21.2 vs. 7.6 min per week, *d* = 0.34, *p* = 0.006) physical activity at three months. The program impacts on moderate physical activity (131.8 vs. 71.0 min per week, *d* = 0.35, *p* = 0.021) and vigorous physical activity (37.7 vs. 8.0 min per week, *d* = 0.26, *p* = 0.040) were sustained among the intervention group at six months. The comparison group showed improvements in light physical activity at three months (150.8 vs. 116.4 min per week, *d* = 0.21, *p* = 0.040), but the effect was not sustained at six months (*p* = 0.108). For sedentary behaviors and all levels of physical activity, larger proportions of participants in the intervention group showed improvements relative to comparison group participants at both three and six months.

### 3.3. Program Effects (Adjusted)

Table 3 presents the immediate and sustained program effects examined using multiple multivariable regression models after controlling for age, sex, race/ethnicity, education, and comorbid conditions, as well as site through random effects modelling. The intervention group showed improvements in social support (adjusted difference = 1.82, *p* < 0.001) and self-efficacy (adjusted difference = 1.07, *p* = 0.003) at 3 months. The differences in the changes from the baseline to three months were significantly different between the intervention and comparison groups (*p* = 0.002).

The intervention group showed a statistically significant reduction in sedentary behaviors (adjusted difference = −0.95, *p* = 0.004) and improvements in light (adjusted difference = 60.36, *p* = 0.014) and moderate (adjusted difference = 62.45, *p* < 0.001) physical activity at three months. The intervention group further showed a reduction in sedentary behavior (adjusted difference = −0.99, *p* = 0.013) and improvements in all levels of physical activity (adjusted difference ranged from 31.01 to 121.55, *p* < 0.05) at six months. For the comparison group, the no significant change was observed at three- or six-month follow-up assessments. The differences in changes from the baseline to three-month follow-up between the intervention and comparison groups were statistically significant for moderate physical activity, with the intervention group showing greater improvements. The differences in changes from baseline to six-month follow-up between the intervention and comparison groups were statistically significant for sedentary behavior as well as all levels of physical activity, with the intervention group showing greater improvements. Figure 2, Figure 3, Figure 4 and Figure 5 present the graphical depiction of changes in sedentary behavior and different levels of physical activity over the three-time points by treatment group.

## 4. Discussion

Findings support our hypotheses that *Texercise Select* participants reported benefits compared to those not enrolled in the intervention. Relative to the comparison group, the intervention group showed greater increases in self-efficacy and perceived social support for engaging in physical activity. Similarly, intervention effects were observed for all types of physical activity and inactivity. Many of the positive gains observed at three months were sustained at six months. While not all participants reported gains for every outcome, about half of the intervention participants or more reported gains for most variables (other than engagement in vigorous activity), which provides additional evidence for the robustness of *Texercise Select* as a lifestyle enhancement program. 

These findings support those previously published from a *Texercise Select* evaluation using a single-group, pre-post study design [26,31] and are consistent with impacts reported in other successful lifestyle interventions for older adults [15,16,17,19]. While the constructs for assessing physical activity were measured differently across the two *Texercise Select* studies, effect sizes for impact on aerobic or moderate physical activity were in a similar range for each construct (i.e., 0.64 for original study and 0.53 for current study). Similarly, effect sizes for the two psychosocial constructs were in a similar range (i.e., 0.45 vs. 0.48 for social support and 0.38 vs. 0.33 for self-efficacy related to physical activity).

In addition to the measures previously examined in *Texercise Select* studies, the current study also assessed sedentary behavior and found significant program impacts. This is a salient finding, given recent studies that demonstrate sedentary behavior as an independent risk factor for poor health outcomes [32]. Interestingly, the intervention also significantly increased light physical activity among participants. This is an important finding especially for older adults who may not be able to engage in more intense activity but can benefit from being more physically active within their functional capacity, as recommended in the national physical activity guidelines [1]. *Texercise Select* is unique in that it is delivered in a community setting (versus a more clinical setting), targets individuals with varied levels of baseline physical activity and physical ability, and is led by trained lay-leaders who utilize a manual specifically tailored to the program with session-by-session guides for education and physical activity intervention components [24]. 

While in other studies [33] there is often a diminution of effect over time, findings from this study showed sustained physical activity at six months or, in some cases improvement over three-month follow-ups (i.e., higher than immediately following the intervention). These improvements over time were significant for the *Texercise Select* participants relative to the comparison group. While immediate improvement measured at the end of the program may include physical activity performed during the class, tracking six-month follow-up assessments enabled us to identify longer-term, sustained program effects (i.e., sustained effects are more impactful than initial effects for lifestyle interventions). As seen in prior literature [26], *Texercise Select* favorably impacted self-efficacy related to physical activity and social support for engaging in physical activity, which may help explain the sustainable increases in physical activity over the six-month assessment period. 

Using the social cognitive learning principles, *Texercise Select* promotes healthy aging behaviors (e.g., physical activity) by enhancing self-efficacy among its participants and encouraging them to take a more active role in their health [24]. The group-based format of *Texercise Select* provides social support and guides its participants to practice correct and safe exercises and use skills, such as goal setting, action planning, and problem-solving [24]. Confirming previous research, it seems that the multiple features of the program helped participants to initiate, engage, and maintain healthy behaviors [12]. Unfortunately, as with many other multi-component interventions, identifying specific features of the program that is effective in promoting physical activity can be challenging. 

### Limitations

There were limitations associated with testing *Texercise Select* in a real-world, community setting designed for service delivery rather than a controlled research environment. As such, the amount and types of measures that could be included were limited to alleviate participant burden. However, to the extent possible, data collection tools were modeled after other community-based surveys with older adults [34]. There are limitations in using self-reported data for identifying changes in physical activity over time and in relation to the program timeline, but these effects may have been lessened because items were used that included narrative prompts and examples, so participants could visualize the different physical activity intensity levels (potentially increasing the accuracy of self-reporting). It is possible that participants were unable to distinguish between different levels of physical activity (i.e., light, moderate, and vigorous) [35]. While the measures identified in the current study were self-reported, we assume that within participant error associated with self-report may be the same from baseline to follow-ups. 

In this quasi-experimental study, randomization was not used to assign implementation sites or participants into study conditions. Group assignment based on feasibility and preference may have introduced imbalance or bias between the study conditions and subsequently influenced the study analyses. Given the pragmatic nature of this study and its implementation in real-world settings, the research team was unable to prevent comparison group participants from participating in some form of exercise (generally or in a formal class structure), other than *Texercise Select*. In this context, this study occurring within real-world conditions allowed us to evaluate program participation versus day-to-day activities that may otherwise be encountered by the target population.

Furthermore, the intervention group who completed the three-month follow-up assessment were less sedentary and engaged in a greater amount of light physical activity, which can limit the generalizability of the study findings. Sensitivity analyses were performed to examine how different baseline levels of sedentary behavior and light physical activity impact the adjusted analyses. Using the median cut-off, adjusted analyses were performed separately among those who reported five or less hours/day of sedentary behavior at the baseline and those who reported more than five hours/day of sedentary behavior at the baseline. Similarly, sensitivity analyses were performed using light physical activity. The sensitivity analyses indicated that baseline levels of sedentary behavior and light physical activity can influence the program effect on sedentary behavior and physical activity. 

Attrition across data collection time points was observed. The reason for dropout for each participant is unknown. However, upon follow-up with program implementers, it became clear that there was no single reason for participants deciding to dropout. Some reasons for dropout expressed by participants to implementers included that the program was not rigorous enough in terms of physical activity, that participants became ill and were unable to complete the program, or that participants had a sickness in the family that prohibited their attendance. Additionally, unforeseen circumstances such as the advent of hurricane-related severe flooding in one of the final control group cities necessitated an abrupt ending to data collection. Additionally, the fixed funding timeline impacted the ability to continue collecting six-month follow-up data on those who entered the study later than originally anticipated. Using site surveys to match site characteristics (ad-hoc) for intervention and comparison group assignment was attempted, however, some significant differences between participants in the study arms were observed. Linear mixed models were conducted adjusting for basic sociodemographic and site factors to help minimize the initial group differences. 

There were no direct measures obtained from participants about program feasibility or acceptability. However, the program is offered free-of-charge to participants in community-based settings and supported by the state, thereby increasing the likelihood of program sustainability. While not directly asked, the vast majority of participants attended at least half of the sessions, which suggests the program content and activities were acceptable and appropriate for most participants. To fully explore program effects on a more generalizable population, future research should explore thresholds of program dose-response effects for a larger, more diverse population, that is inclusive of more middle-aged and older adults, and in different geographic regions. 

## 5. Conclusions

These findings are encouraging because they indicate the positive effects of *Texercise Select* on physical activity and related outcomes for older adults. Further, they demonstrate that older adults are interested in attending a multi-modal lifestyle intervention program and are able to make health behavior changes that can improve health and overall functioning. While there are other physical activity-oriented, evidence-based programs for older adults, the proportion of older adults enrolling in these programs is small relative to the large number of older adults needing intervention and intervention-related benefits. Therefore, *Texercise Select* is a welcomed addition to effective physical activity and wellness programs that can serve older adults to improve health and wellbeing. Additionally, given the interventions name brand and lasting history in Texas [24], *Texercise Select* has some unique advantages for attracting older adults in Texas. Having the program embedded within a state agency is invaluable in achieving program scalability and sustainability. Given the findings, we recommend that this program be promoted throughout the aging services sector to serve as a complement to both existing behavioral intervention (e.g., focused on management of chronic disease [36], falls [37]) and other types of interventions (e.g., designing walkable communities [38,39]).

## Figures and Tables

**Table 1:** Baseline characteristics of the study participants (Intervention vs. Comparison).

	Descriptive Statistics ^a^			Descriptive Statistics ^a^			Descriptive Statistics ^a^		
	Overall Baseline			Matching Baseline and 3-Month Follow-Up			Matching Baseline and 6-Month Follow-Up		
	Intervention (*n* = 163)	Comparison (*n* = 267)	*p*-Value ^b^	Intervention (*n* = 126)	Comparison (*n* = 173)	*p*-Value ^c^	Intervention (*n* = 74)	Comparison (*n* = 108)	*p*-Value ^d^
Age (years)	74.8 (7.70)	74.3 (9.65)	0.505	74.6 (7.81)	74.4 (9.16)		73.7 (7.54)	75.2 (7.91)	
Female	129 (79.1%)	204 (76.4%)	0.510	102 (81.0%)	137 (79.2%)	0.707	58 (78.4%)	83 (76.9%)	0.809
Non-Hispanic White	105 (67.7%)	87(34.7%)	<0.001	79 (65.8%)	59 (36.2%)	<0.001	45 (63.4%)	36 (35.0%)	<0.001
Education			0.058			0.533			0.338
High school graduate or lower	56 (34.4%)	110 (41.4%)		46 (36.5%)	73 (42.4%)		27 (36.5%)	50 (46.7%)	
Some college	62 (38.0%)	72 (27.1%)		43 (34.1%)	50 (29.1%)		28 (37.8%)	31 (29.0%)	
College graduate or higher	45 (27.6%)	84 (31.6%)		37 (29.4%)	49 (28.5%)		19 (25.7%)	26 (24.3%)	
Having 2 or more chronic conditions	111 (68.5%)	166 (65.1%)	0.471	87 (69.6%)	111 (66.9%)	0.621	52 (71.2%)	72 (71.3%)	0.994
Sedentary (h/day)	5.7 (3.35)	5.4 (3.60)	0.322	5.4 (3.24)	5.5 (3.39)	0.799	5.4 (2.82)	5.3 (3.11)	0.994
Light (min/week)	156.0 (212.46)	118.8 (120.93)	0.047	172.6 (234.75)	115.0 (117.03)	0.015	182.8 (263.27)	119.7 (99.31)	0.057
Moderate (min/week)	71.7 (107.68)	78.7 (143.57)	0.576	74.6 (114.33)	71.0 (127.71)	0.803	70.7 (98.49)	78.4 (144.75)	0.698
Vigorous (min/week) ^e^	7.9 (25.84) 0 [0, 0]	16.0 (46.81) 0 [0, 0]	0.175	7.4 (23.07) 0 [0, 0]	15.1 (47.48) 0 [0, 0]	0.532	9.2 (24.67) 0 [0, 0]	18.8 (55.58) 0 [0, 0]	0.890
Social support for physical activity	9.0 (3.40)	9.4 (3.48)	0.245	9.4 (3.46)	9.6 (3.44)	0.630	9.4 (3.28)	9.7 (3.25)	0.635
Self-efficacy for physical activity	18.5 (3.49)	17.7 (3.78)	0.022	18.4 (3.61)	17.7 (3.77)	0.167	18.2 (3.50)	17.5 (3.77)	0.237

^a^ = Mean (SD), Freq (%), or Median (IQR); ^b^ = *p*-values from the univariate analysis between intervention and comparison groups among all study-eligible participants; ^c^ = *p*-values from the univariate analysis between intervention and comparison groups among all study-eligible participants with matched pre- and post-test surveys at 3 months; ^d^ = *p*-values from the univariate analysis between intervention and comparison groups among all study-eligible participants with matched pre- and post-test surveys at 6 months; ^e^ = Among the overall study-eligible participants, 87.0% in intervention and 82.5% in comparison groups reported not engaging in any vigorous physical activity. Excluding those who reported 0, the mean (SD) were 60.6 (44.91) for intervention group and 93.0 (75.31) for comparison group.

**Table 2:** Unadjusted immediate and sustained program effects on engagement in physical activity.

	Immediate Program Effects:Compared Baseline with 3-Month Follow-Up Assessment					Sustained Program Effects:Compared Baseline with 6-Month Follow-Up Assessment				
	Means (SD)		% of Those Who Showed Improvements	Effect Size(95% CI)	*p*-Values	Means (SD)		% of Those Who Showed Improvements	Effect Size(95% CI)	*p*-Values
	Baseline	3-Month Follow-Up				Baseline	6-Month Follow-Up			
N										
Comparison	173	173	-	-	-	108	108	-	-	-
Intervention	126	126	-	-	-	74	74	-	-	-
Sedentary (h/day)										
Comparison	5.5 (3.44)	5.1 (3.08)	41.4%	0.13(−0.03, 0.29)	0.108	5.3 (3.11)	5.4 (3.79)	46.1%	−0.02(−0.21, 0.18)	0.878
Intervention	5.4 (3.24)	4.8 (2.82)	52.5%	0.20(−0.02, 0.35)	0.079	5.4 (2.82)	4.8 (2.88)	50.0%	0.21(−0.06, 0.43)	0.129
Light PA (min/week)										
Comparison	116.4 (118.41)	150.8 (197.60)	53.6%	0.21(0.01, 0.33)	0.040	122.5 (99.22)	147.3 (172.50)	46.5%	0.18(−0.04, 0.36)	0.108
Intervention	174.6 (237.14)	226.3 (290.50)	57.8%	0.19(−0.03, 0.33)	0.107	182.0 (269.05)	278.0 (422.57)	61.8%	0.27(−0.05, 0.43)	0.119
Moderate PA (min/week)										
Comparison	74.8 (131.74)	89.4 (141.79)	35.7%	0.11(−0.04, 0.28)	0.147	78.4 (144.75)	81.1 (93.97)	43.3%	0.02(−0.17, 0.21)	0.842
Intervention	70.7 (107.83)	128.5 (109.06)	66.1%	0.53(0.36, 0.72)	<0.001	71.0 (99.16)	131.8 (227.71)	47.9%	0.35(0.04, 0.52)	0.021
Vigorous PA (min/week)										
Comparison	15.7 (48.38)	17.7 (54.91)	16.5%	0.04(−0.12, 0.19)	0.548	18.9 (55.81)	21.3 (69.57)	15.2%	0.04(−0.16, 0.22)	0.924
Intervention	7.6 (23.34)	21.2 (52.05)	22.8%	0.34(0.08, 0.45)	0.006	8.0 (23.66)	37.7 (159.20)	23.1%	0.26(−0.06, 0.43)	0.040
Social support for engaging in PA ↑										
Comparison	9.6 (3.45)	10.2 (3.30)	45.1%	0.16(0.01, 0.33)	0.032	NA	NA	NA	NA	NA
Intervention	9.4 (3.47)	11.0 (3.19)	58.8%	0.48(0.25, 0.62)	<0.001	NA	NA	NA	NA	NA
Self-efficacy to engage in PA ↑										
Comparison	17.8 (3.72)	18.1 (3.57)	37.7%	0.07(−0.09, 0.23)	0.371	NA	NA	NA	NA	NA
Intervention	18.4 (3.61)	19.5 (3.19)	49.2%	0.33(0.07, 0.43)	0.008	NA	NA	NA	NA	NA

SD = Standard deviation; CI = Confidence interval; ↑ = higher value indicates better (e.g., greater social support or higher self-efficacy).

**Table 3:** Adjusted immediate and sustained program effects on engagement in physical activity.

Health Behaviors	Adjusted Means (95% CI)			Adjusted Differences (95% CI), Changes from Baseline		*p*-Values (Changes from Baseline to Immediate Post or 6-Month Post-Test)		*p*-Values (“Group × Time” Interaction)
	T1. Baseline	T2. 3-Month Follow-Up	T3. 6-Month	Immediate Program Effects: Compare T1 vs. T2	Sustained Program Effects: Compare T1 vs. T3	Immediate Program Effects: Compare T1 vs. T2	Sustained Program Effects: Compare T1 vs. T3	
Sedentary (h/day)								0.079 ^a^ 0.035 ^b,^*
Comparison	5.28 (4.85, 5.72)	5.10 (4.57, 5.63)	5.40 (4.75, 6.04)	−0.18 (−0.74, 0.38)	0.11 (−0.55, 0.78)	0.525	0.735	
Intervention	5.47 (4.94, 6.00)	4.52 (3.94, 5.10)	4.48 (3.77, 5.18)	−0.95 (−1.60, −0.30)	−0.99 (−1.78, −0.21)	0.004	0.013	
Light PA (min/week)								0.397 ^a^ 0.019 ^b,^*
Comparison	122.54 (93.79, 151.30)	155.67 (119.83, 191.50)	153.74 (110.83, 196.65)	33.12 (−7.72, 73.96)	31.19 (−15.90, 78.29)	0.112	0.194	
Intervention	150.11 (110.05, 190.17)	210.46 (166.45, 254.48)	271.66 (216.47, 326.85)	60.36 (12.13, 108.59)	121.55 (62.81, 180.30)	0.014	<0.001	
Moderate PA (min/week)								0.008 ^a,^* 0.003 ^b,^*
Comparison	71.73 (54.67, 88.80)	89.13 (68.13, 110.14)	74.24 (49.71, 98.77)	17.40 (−4.40, 39.21)	2.51 (−22.67, 27.68)	0.118	0.845	
Intervention	73.40 (53.29, 93.51)	135.75 (113.40, 158.11)	135.85 (107.69, 164.01)	62.35 (37.10, 87.61)	62.45 (32.20, 92.70)	<0.001	<0.001	
Vigorous PA (min/week)								0.116 ^a^ 0.004 ^b,^*
Comparison	17.68 (9.44, 25.92)	16.69 (6.48, 26.91)	17.02 (4.57, 29.46)	−0.98 (−12.53, 10.57)	−0.66 (−14.21, 12.89)	0.867	0.924	
Intervention	13.77 (3.46, 24.08)	27.15 (15.86, 38.45)	44.78 (30.38, 59.18)	13.38 (−0.34, 27.11)	31.01 (14.63, 47.39)	0.056	<0.001	
Social support for engaging in PA ↑								0.002 ^a,^*
Comparison	9.09 (8.63, 9.56)	9.59 (9.04, 10.14)	NA	0.50 (−0.03, 1.03)	NA	0.065	NA	
Intervention	9.40 (8.83, 9.97)	11.22 (10.59, 11.84)	NA	1.82 (1.19, 2.44)		<0.001		
Self-efficacy to engage in PA ↑								0.098 ^a^
Comparison	18.06 (17.55, 18.56)	18.36 (17.77, 18.94)	NA	0.30 (−0.29, 0.90)	NA	0.320	NA	
Intervention			NA	1.07 (0.38, 1.76)		0.003		

PA = Physical activity; CI = Confidence Interval; Adjustment = All models were controlled for age, sex, race/ethnicity, education, and chronic comorbidity; * = Intervention group showed a greater improvement over time than the comparison group; ^a^ = Statistical significance of differences in changes from the baseline to 3 months follow-up between the intervention and comparison groups; ^b^ = Statistical significance of differences in changes from the baseline to 6 months follow-up between the intervention and comparison groups; ↑ = higher value indicates better (e.g., greater social support or higher self-efficacy).
